# Convulsions in Children

**Published:** 1875-02

**Authors:** Wm. T. Beall

**Affiliations:** Edwards, Mississippi


### CONVULSIONS IN CHILDREN.

BY WM. T. BEALL, M.D., EDWARDS, MISSISSIPPI.

I would like to offer a few remarks upon the treatment of convulsions in children, as I have never seen anything on the subject in keeping with my experience.

I believe it has always been the idea that the first and most important thing to be done in infantile convulsions from teething, worms, or any other cause, is to bathe the patient in hot mustard water; but an accidental circumstance many years ago taught me that counter-irritation to the spine, by means of turpentine or chloroform, is far more readily applied, and more certain to afford relief. In fact, an experience of fifteen years, during which time I have had a great many cases, particularly in that season of the year when remittent fever prevails, has so thoroughly convinced me of the superiority of this treatment, that I never use the hot bath in such cases, but rely upon counter-irritants to the spine, stimulating enemas, and in the interval quinine, if it be a case of malarial fever.

I do not offer this as anything new, but am induced to write because I have never seen the idea advanced in any of the journals. The spinal application of chloroform is also efficient in puerperal convulsions.

If the following article is worth mention, please insert it in the RECORD:

*CHOLERA INFANTUM ALIAS MISPLACED CHILL.*

*Case:* Child 12 months old; taken at one o'clock at night with vomiting and purging, which lasted several hours, followed by slight fever. At one o'clock the next night, same symptoms appeared, and again followed by fever. *Treatment:* Broken doses of calomel and morphine; quinine every two hours, beginning eight hours before the next exacerbation. *Diet:* Fresh cow's milk, weakened with water. Recovery rapid after use of quinine.

---

PHLEGMASIA DOLENS.—Dr. Carson says of one case: "I determined to avoid the usual treatment laid down in the books, because I had never seen the slightest good result from it. I put the lady permanently in bed, gave nourishment freely in a simple form, and three or four glasses of wine in twenty-four hours. There was no local application whatever, nor was there any internal medicine, except as hereafter stated. Some years since, my friend, Dr. Henry Purdon, of Belfast, told me of the success he had had in treating puerperal peritonitis by the bisulphite of soda. I have lately used this medicine in various forms of fever, and other diseases, with marked advantage. I therefore prescribed it in this case of phlegmasia dolens. The dose was twenty grains, in solution, every third hour.

"In twenty-four hours the constitutional symptoms became visibly improved. In three days the pain and swelling began to subside, and were totally gone in five days. The lochia, at the end of that time, returned, and the milk became abundant. The patient was able to rise, and has been perfectly well ever since."—*London Medical Times and Gazette.*

